# The BCMA-Targeted Fourth-Generation CAR-T Cells Secreting IL-7 and CCL19 for Therapy of Refractory/Recurrent Multiple Myeloma

**DOI:** 10.3389/fimmu.2021.609421

**Published:** 2021-03-05

**Authors:** Deming Duan, Keke Wang, Cheng Wei, Dudu Feng, Yonghua Liu, Qingyan He, Xing Xu, Chunling Wang, Shuping Zhao, Leili Lv, Jing Long, Danni Lin, Ai Zhao, Bingmu Fang, Jinhong Jiang, Shixing Tang, Jimin Gao

**Affiliations:** ^1^Guangdong Provincial Key Laboratory of Tropical Disease Research, School of Public Health, Southern Medical University, Guangzhou, China; ^2^Wenzhou Institute, University of Chinese Academy of Sciences, Wenzhou, China; ^3^Key Laboratory of Laboratory Medicine, Ministry of Education, School of Laboratory Medicine and Life Sciences, Wenzhou Medical University, Wenzhou, China; ^4^Department of Hematology, Shunde Hospital, Southern Medical University, Foshan, China; ^5^Department of Hematology, Lishui People's Hospital, Lishui, China; ^6^Harvard Medical School, Boston, MA, United States; ^7^Zhejiang Qixin Biotech, Wenzhou, China

**Keywords:** multiple myeloma, CAR-T, BCMA, IL-7, CCL19

## Abstract

Chimeric antigen receptor (CAR) technology has revolutionized cancer treatment, particularly in malignant hematological tumors. Currently, the BCMA-targeted second-generation CAR-T cells have showed impressive efficacy in the treatment of refractory/relapsed multiple myeloma (R/R MM), but up to 50% relapse remains to be addressed urgently. Here we constructed the BCMA-targeted fourth-generation CAR-T cells expressing IL-7 and CCL19 (i.e., BCMA-7 × 19 CAR-T cells), and demonstrated that BCMA-7 × 19 CAR-T cells exhibited superior expansion, differentiation, migration and cytotoxicity. Furthermore, we have been carrying out the first-in-human clinical trial for therapy of R/R MM by use of BCMA-7 × 19 CAR-T cells (ClinicalTrials.gov Identifier: NCT03778346), which preliminarily showed promising safety and efficacy in first two enrolled patients. The two patients achieved a CR and VGPR with Grade 1 cytokine release syndrome only 1 month after one dose of CAR-T cell infusion, and the responses lasted more than 12-month. Taken together, BCMA-7 × 19 CAR-T cells were safe and effective against refractory/relapsed multiple myeloma and thus warranted further clinical study.

## Introduction

Multiple myeloma (MM) is characterized by the abnormal expansion of bone marrow plasma cells (1). Despite the advent of new therapies such as monoclonal antibodies, immunomodulatory drugs, and autologous stem cell transplant, MM remains incurable (2, 3). Treating patients with refractory/relapsed multiple myeloma (R/R MM) is challenging, and new treatments are critically needed.

In recent years, chimeric antigen receptor (CAR)-T cell immunotherapy has made outstanding advances in the treatment of B-cell malignant hematological tumors (4–9). CARs are synthetic fusion molecules, mostly single-chain fragment variables (scFvs) derived from a monoclonal antibody (mAb) (10). CAR-T cells can specifically recognize surface molecules on target cells independent of major histocompatibility complex (MHC)-peptide presentation and subsequently induce target cell lysis through the release of perforin and granzyme B (11). Fourth-generation CAR-T cells, known as armored CAR-T cells, co-express key cytokines, such as interleukins and chemokines, or suicide genes that can significantly enhance the efficacy and safety of CAR-T therapy (1, 12, 13).

B-cell maturation antigen (BCMA) (14–16) is a cell surface protein expressed in tumor cells and involved in the maturation and differentiation of B cells into plasma cells. BCMA is highly expressed in malignant MM plasma cells but rarely found in normal tissue, except normal plasma cells (17, 18), which makes it a promising target for BCMA-directed immunotherapy. Previous clinical studies have shown efficacy against MM (19); however, a lack of durable effector functions by conventional CAR-T cells lead to up to 50% relapse (15, 20). Further studies have to be conducted urgently to determine the optimal CARs for treating MM.

To decrease MM relapse, we developed a fourth-generation BCMA-targeted CAR-T secreting IL-7 and CCL19 for R/R MM with superior long-term effector functions. Studies have indicated that IL-7 and CCL19 play important roles in the maintenance and formation of the T-zone in lymphoid organs (21, 22). IL-7, a non-hematopoietic cell-derived cytokine critical for the development of the immune system, is a major regulator of proliferation and homeostasis of CD8 and CD4 T cells (23–25). CCL19, which is constitutively expressed by stromal cells in the lymphoid T-zone (26, 27), is a chemotactic agent for dendritic cells and T cells migrating to secondary lymphoid tissue and plays an important role in the initiation of the adaptive immune response (28).

Here we demonstrated that BCMA-7 × 19 CAR-T cells are capable of eradicating MM cells both *in vitro* and *in vivo* and showed preliminarily their promising safety and efficacy in the first two enrolled patients of our ongoing first-in-human clinical trial.

## Materials and Methods

### Culture Conditions

MM1S, U266, and K562 cells, purchased from ATCC, were maintained in RMPI-1640 medium (Sigma) and HEK-293T cells were maintained in DMEM(Sigma) supplemented with 10% FBS (PAN), 1% sodium pyruvate (Gibco), 1% L-glutamine (Gibco) and 1% Pen Strep (Gibco). All cells were cultured under 5% CO_2_ at 37°C and were routinely tested for mycoplasma contamination.

### Isolation and Transduction of T Cells

Peripheral blood mononuclear cells (PBMCs) were harvested from healthy donors or patients and isolated by density gradient centrifugation. T cells were enriched and activated by anti-CD3/CD28 coated beads (Invitrogen) and cultured in X-VIVO serum-free medium (Lonza, 04-744Q) supplemented with 5% AB serum (Sigma), 10% nonessential amino acids (Corning), 0.01% recombinant human IL-2, and 0.05% IL-7 and IL-15 (PeproTech). Transduction was performed with CAR-encoding lentiviral vector after 24 h of stimulation, and lentiviral transduction efficiency was assessed 7 days after transduction by flow cytometry.

### Flow Cytometry

FITC-labeled BCMA protein (ACRO) and PE-conjugated anti-BCMA antibody were used to detect lentiviral transduction efficiency and verify expression of the target antigen on tumor cells. For intracellular staining, we used the Cytofix/Cytoperm Kit (BD Biosciences) to fix and permeabilize cells, then labeled the cells with APC-conjugated anti-IFN-γ antibody and APC-conjugated anti-IL-2 antibody. Anti-CD62L antibody (PE), anti-CCR7 antibody (PerCP-Cy5.5), anti-CD45RO antibody (FITC), anti-CD45RA antibody (APC), anti-CD8α antibody (PE-Cy7), and anti-CD4 antibody (APC-Cy7) were used to stain surface markers on T cells. All antibodies of brands not mentioned above were from BioLegend. Data were acquired with a BD FACS AriaII (BD Biosciences) and analyzed with FlowJo X (FlowJo).

### Cytokine Assays

Enzyme-linked immunosorbent assay (ELISA) was performed to detect and quantify concentrations of soluble cytokine and chemokine proteins. The culture supernatant of CAR-T cells was retained 3 and 5 days after transduction, and levels of IL-7 and CCL19 were analyzed with an IL-7 ELISA kit (R&D Systems) and CCL19 ELISA kit (NeoBioscience), respectively. Effector cells were co-cultured with MM1S (1 × 10^5^ cells/well) at a 1:1 ratio. After 24 h, a GM-CSF ELISA kit (BD Biosciences) was used to measure the concentration of GM-CSF in the culture supernatant.

### Transwell Migration Assay

Chemotaxis on T cells was measured with a transwell (Corning) with a 5 μm pore permeable membrane insert. The transwell chamber was placed in a 24-well plate (BIOFIL). CFSE-labeled T cells were seeded in the upper chamber, and the 5-day supernatant of the CAR-T cell culture was added to the lower chamber. The cells were incubated at 37°C and 5% CO_2_, and the number of cells that migrated from the upper to the lower chamber was evaluated under a fluorescence microscope.

### Cellular Cytotoxicity Assays

Bioluminescence assays of luciferase were performed to determine cytotoxic activity of CAR-T cells against BCMA-expressing target cells. 1 × 10^4^ target cells were co-incubated in a flat-bottomed 96-well tissue culture plate (BIOFIL) for 4 h with CAR-T cells at various E:T ratios. Untransduced cells served as a negative control. One group in which only target cells and RPMI1640 medium were added was set as the maximum value (Max), and another group that contained target cells and ddH_2_O was set as the minimum background value (Min). Each group consisted of three auxiliary holes. Specific lysis was calculated as follows: lysis (%) = (Max – V)/(Max – Min) × 100%.

### *In vivo* Analysis of CAR-T Activity

NSG mice were purchased from GemPharmatech and injected intravenously with 4 × 10^6^ BCMA-K562-Luc-GFP cells on day 0. Mice were randomly divided into two cohorts (*n* = 3 per cohort). Then 6 × 10^6^ BCMA-7 × 19 CAR-T cells (experimental group) and mock-T cells (control group) were injected intravenously on day 7. Tumor burden was measured by intraperitoneal injection of 150 mg/kg D-luciferin and imaging 2 min later with an exposure time of 30 s by the IVIS® Spectrum BL. Living Image was used to assess bioluminescence for each mouse as photons/s/cm^2^/sr. Imaging was performed on days 7, 10, 17, and 24 to monitor tumor progression. All reagents and instruments not annotated above were from PerkinElmer.

### Statistical Analysis

All statistical analyses were performed with GraphPad Prism v6.0. The data are shown as the mean ± SD (*N* = 3). Two-way analysis of variance (ANOVA) by multiple comparisons test and the two-tailed unpaired *t*-test were used for comparison of 3 or 2 groups, respectively. Differences at *P*-values < 0.05 were considered significant.

### Study Design and Participants

This study reports early clinical experience from the Sixth Affiliated Hospital of Wenzhou Medical University. The protocol is included in **Figure 4B**. Enrolled patients were 18–80 years old with a confirmed diagnosis of R/R MM as defined by International Myeloma Working Group criteria (29). All patients provided written informed consent before treatment. The study was approved by the Ethics Committee of the Sixth Affiliated Hospital of Wenzhou Medical University.

### Assessments

AEs were identified and graded according to the Common Terminology Criteria for Adverse Events (CTCAE) v5.0. CRS was assessed with the modified criteria proposed by Lee et al. (30). We assessed CR and VGPR according to International Myeloma Working Group (IMWG) updated diagnostic criteria for multiple myeloma in this clinical trial (31, 32)

## Results

### Generation and Characterization of BCMA-7 × 19 CAR-T Cells

**Figure 1:**
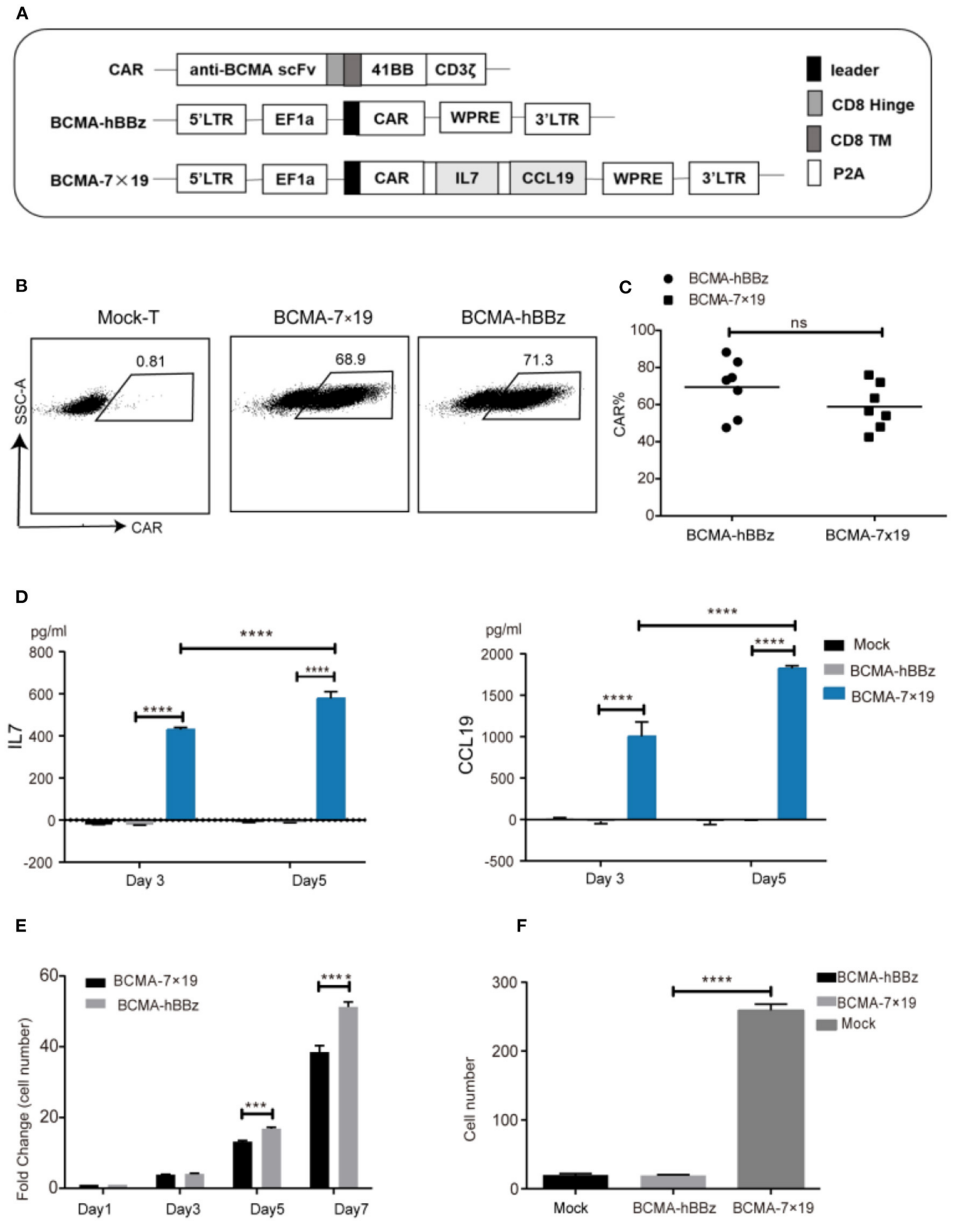
Efficient expression of CAR on lentiviral-transduced T cells and functional verification of IL-7 and CCL19. **(A)** Schematic representation of BCMA-hBBz CAR and BCMA-7x19 CAR. **(B)** CAR expressions of CAR-T cells were analyzed by flow cytometry. The numbers in scatter plots represent the percentages of positively stained cells. **(C)** The expression rates of BCMA CAR and BCMA-7x19 CAR. Data are means± SD obtained from seven donors. MOI = 80. *P*-value was calculated by two-tailed student *t*-test. ns, not statistically significant (*P* > 0.05). **(D)** Quantitative detection of IL-7 (left) and CCL19 (right) secretion by ELISA. **(E)** Number fold change of BCMA-hBBz and 7 × 19 CAR-T cells at the indicated time-points. ****P < 0.0001 by two-way ANOVA. **(F)** The count of migrating T cells (*N* = 3). ****P < 0.0001 was calculated by two-tailed student *t*-test. ***P < 0.001.

We constructed the plasmids carrying the second-generation CAR (BCMA-hBBz), which contained the anti-BCMA scFv, the CD8 transmembrane region, and the intracellular signaling domains of human 4-1BB and the CD3ζ motif in tandem. A 2A linker sequence was inserted directly downstream of CARs followed by IL-7 and CCL19 molecules for equal molar expression, designated BCMA-7 × 19 CAR (Figure 1A). Flow cytometric data showed high transduction efficiency in both BCMA-hBBz CAR-T and BCMA-7 × 19 CAR-T on day 5 after transduction (Figures 1B,C).

We next validated the functional secretion of IL-7 and CCL19 separately. As shown in Figure 1D, significantly increased concentrations of IL-7 and CCL19 were observed in BCMA-7 × 19 CAR-T cells compared to mock-T and BCMA-hBBz CAR-T cells on days 3 and 5. In line with the facts that IL-7 enhances the proliferation of T cells and CCL19 is a chemoattractant for CCR7^+^ T cells (23, 24, 28, 33), we examined the absolute number of cells and performed transwell migration assay. The results demonstrated that IL-7 secreted by BCMA-7 × 19 CAR-T cells enhanced the proliferation and survival of CAR-T cells, and CCL19 promoted lymphocyte migration and recruitment of peripheral T lymphocytes (Figures 1E,F).

### An Extraordinarily High Proportion of Stem Cell-Like Memory T cells (Tscm)

**Figure 2:**
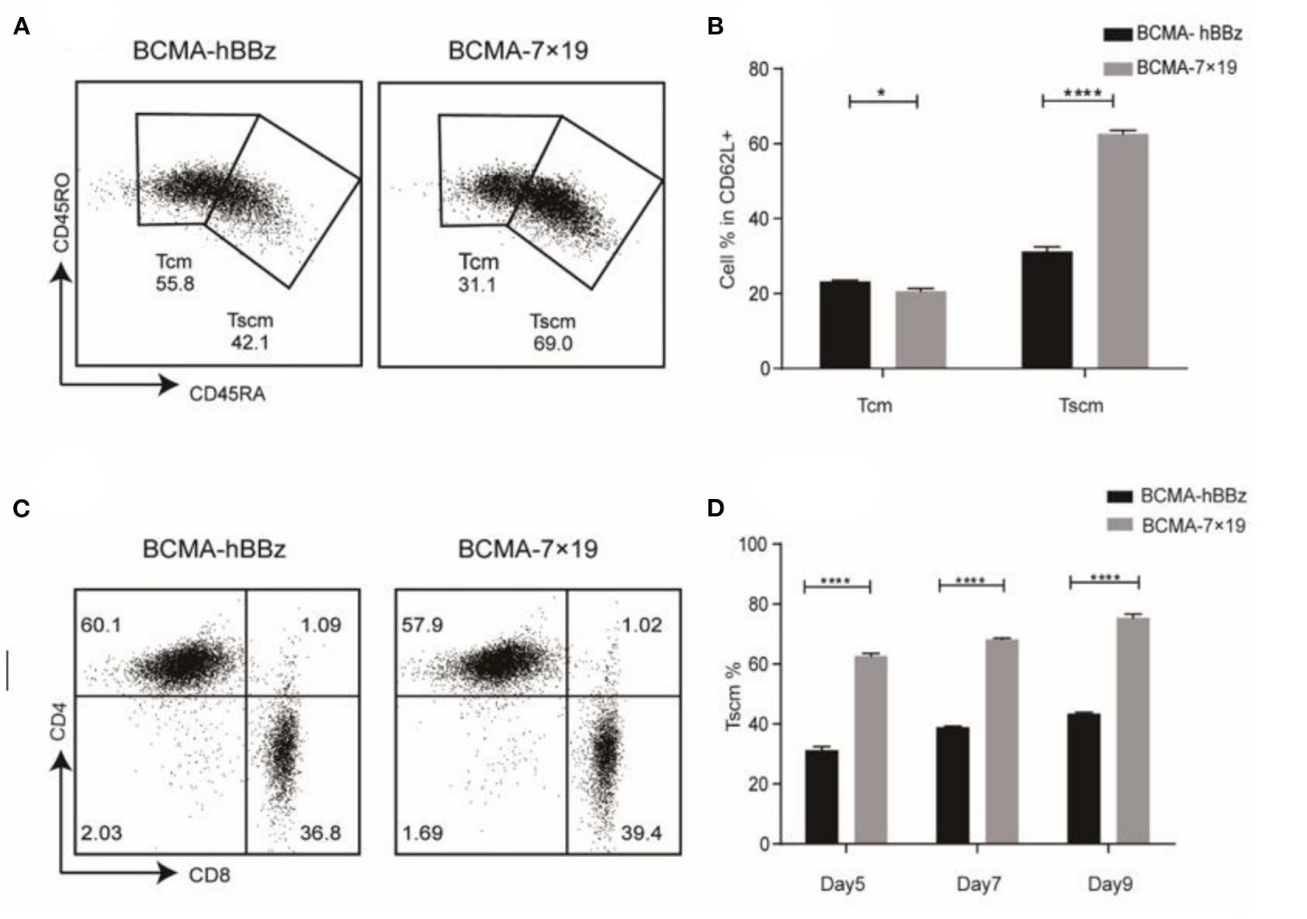
Phenotype of CAR-T cell. **(A)** Expression of CD45RA and CD45RO on CAR-T cells subsets in CD62L^+^ cells by flow cytometry. Tcm (CD62L^+^CD45RO^+^CD45RA^−^), Tscm (CD62L^+^CD45RO^−^CD45RA^+^). **(B)** Statistical chart of subset distribution. **(C)** CD4/CD8 ratios of CAR-T cells were analyzed by flow cytometry. **(D)** Change of Tscm ratio at the indicated time-points. *P*-value was calculated by two-way ANOVA. *P < 0.05, ****P < 0.0001.

Naïve T cells differentiate into Tscm, central memory T cells (Tcm), effector memory T cells (Tem), and effector T cells (Teff). Preclinical models revealed that Tscm—defined by the expression of CD45RA, CD45RO, and CD62L—had greater potential for self-renewal and pluripotent differentiation, longer persistence and greater anti-tumor activity compared to Tcm (34–36). BCMA-7 × 19 CAR-T cells showed a higher Tscm ratio by flow cytometry due to delayed terminal differentiation (Figures 2A,B). The CD4/CD8 ratio showed no significant difference (Figure 2C). We also monitored changes in the Tscm ratio at 5, 7, and 9 days after transduction. Results showed that the durable and effectiveness of BCMA-7 × 19 CAR-Tscm vs. Tcm was time-independent, indicating that it probably had a higher and longer anti-tumor potential (Figure 2D).

### Cytotoxicity of BCMA-7 × 19 CAR-T Cells *in vitro* and *in vivo*

**Figure 3:**
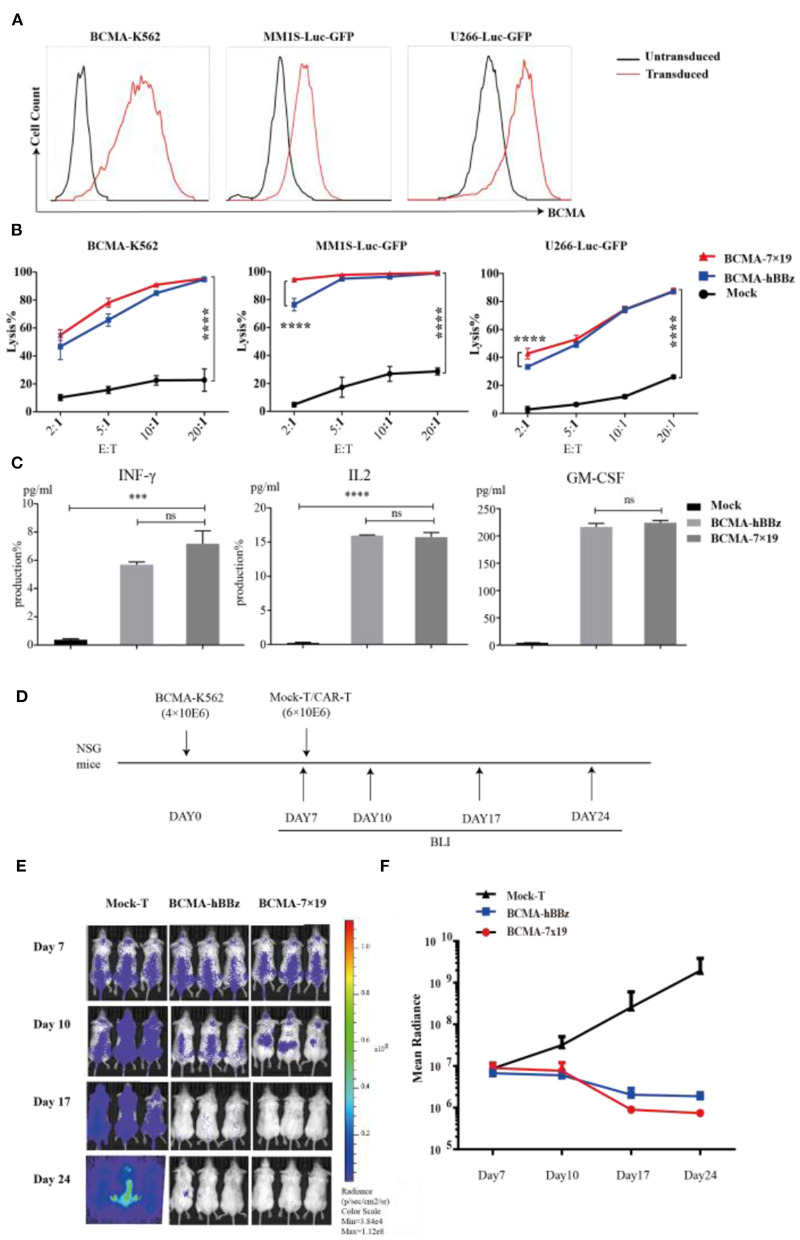
Cytotoxicity analysis of BCMA-hBBz and 7 × 19 CAR-T cells *in vitro* and *in vivo*. **(A)** MM1S-Luc-GFP, U266-Luc-GFP and BCMA-K562 cell lines stably expressing BCMA and luciferase. **(B)** CAR-T cells and target tumor cells were co-incubated for 4 h at the indicated E:T ratios. Cytotoxicity assay with BCMA-K562 (left), MM1S-Luc-GFP (middle) and U266-Luc-GFP cells as targets (right). Differences between groups were determined using two-way ANOVA. Mean ± SD, ****p < 0.0001. **(C)** Cytokine release by CAR-T cells in response to multiple myeloma cell lines. CAR-T or mock-T cells were incubated with MM1S-Luc-GFP cells at 1:1 for 24 h, IL2 (left), IFN-γ (middle) and GM-CSF (right) were analyzed by intracellular staining or ELISA. *P*-value was calculated by two-tailed student *t*-test. ***P < 0.001. ns, not statistically significant (*P* > 0.05). **(D)** Flow chart of animal experimentation. **(E)**. On day 0, NSG mice were injected intravenously with 4 × 10^6^ BCMA-K562 cells. On day 7, mice received 6 × 10^6^ BCMA-7 × 19 CAR-T cells (*n* = 3), BCMA-hBBz CAR-T cells (*n* = 3) or mock-T cells (*n* = 3). Luciferase bioluminescent imaging analysis on days 7, 10, 17, and 24. **(F)** Average bioluminescent signal for each group in different days [mean radiance (p/s/cm^2^/sr)] ±SD.

To determine the anti-tumor activity of BCMA-7 × 19 CAR-T cells *in vitro*, we generated U266-Luc-GFP, MM1S-Luc-GFP, and BCMA-K562-Luc-GFP cells, which could simultaneously express the target antigen and luciferase (Figure 3A). We next determined cytotoxicity by co-culturing T cells with the three target cells. BCMA-hBBz CAR-T and BCMA-7 × 19 CAR-T cells specially and effectively lysed the BCMA-expressing cell lines, whereas mock-T cells showed a background killing at various effector-to-target cell (E:T) ratios. It needs to be emphasized that BCMA-7 × 19 CAR-T cells showed significantly stronger cytotoxicity compared to BCMA-hBBz CAR-T cells at low E:T ratios (Figure 3B). Moreover, we verified similar levels of IL-2, IFN-γ, and GM-CSF released by CAR-T cells after specific killing, which were strongly associated with neurotoxicity and cytokine release syndrome (CRS; Figure 3C).

Subsequently, we assessed the anti-tumor effects of BCMA-7 × 19 CAR-T cells *in vivo* (Figure 3D). NSG mice were inoculated with 4 × 10^6^ BCMA-K562-Luc-GFP cells intravenously on day 0 as a xenogeneic model of MM. On day 7, the xenogeneic mice were treated with an intravenous injection of 6 × 10^6^ BCMA-7 × 19 CAR-T cells, BCMA-hBBz CAR-T cells or untransduced mock-T cells. As shown in Figures 3E,F, the mice treated with BCMA-7 × 19 CAR-T cells displayed a significant decrease in systemic tumor burden as evidenced by signal intensity 17 days after cell infusion. By contrast, luciferase activity rapidly and steadily increased in mice injected with the mock-T cells. Taken together, our data showed that BCMA-7 × 19 CAR-T cells efficiently and markedly lysed tumor cells expressing BCMA on the surface *in vivo* and *ex vivo*.

### Demographics and Baseline Characteristics

**Figure 4:**
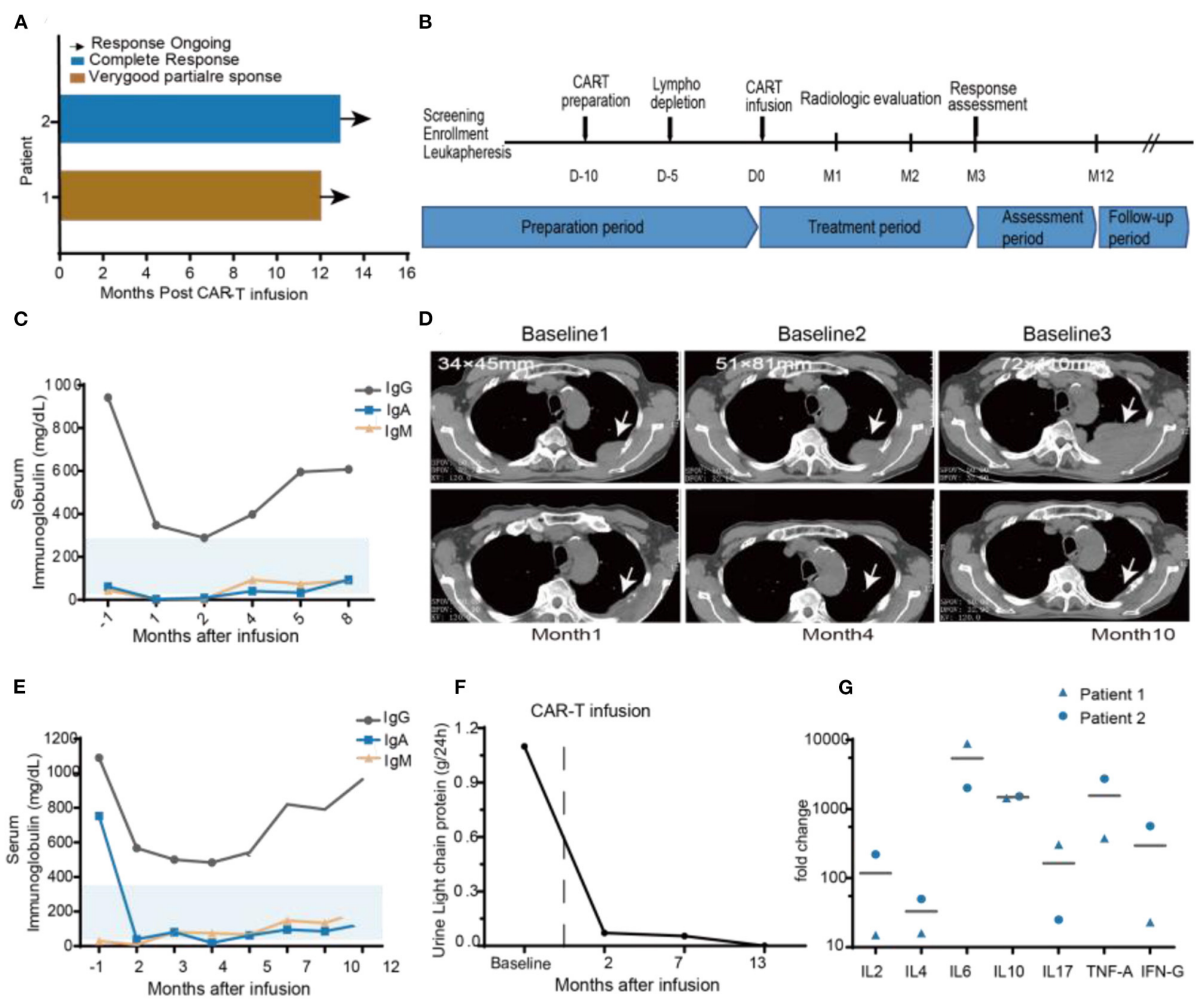
Clinical efficacy after BCMA-7 × 19 CAR-T infusion. **(A)** Duration of response and survival after BCMA-7X19 CAR-T cell infusion. **(B)** Scheme for CAR-T preparation and treatment. **(C)** Patient 1, serum immunoglobulin IgG, IgA, IgM are in the normal range, both before and after infusion. **(D)** Patient 1, representative CT scans at different baselines (baseline1: First extramedullary recurrence of multiple myeloma; 1 year after Len and DXM induction therapy shown in baseline 2: progression of extramedullary recurrence; baseline 3: 1 month before BCMA-7X19 CAR-T infusion); and CT scans post-CAR-T cell infusion 1, 4, and 10 months. Scale bars, 1 cm. **(E)** Patient 2, monitoring of urine light chain protein and **(F)** Serum immunoglobulin IgG, Ig A, Ig M levels before and after treatment. **(G)** Serum kinetics of a panel of cytokines in patients who received infusions with high doses of CAR-T cells, as determined by Luminex multiplex assay (R&D Systems) Horizontal lines denote mean values.

We obtained the following results from two R/R MM patients showing the safety and efficacy of BCMA-7 × 19 CAR-T cell therapy. The patients had received three to five prior lines of treatment but eventually developed R/R MM. The clinical baseline characteristics of the two subjects were summarized in Table 1. Patient 1, a 69-year-old man, presented with a soft tissue mass in the left fourth rib about 72 × 110 mm and progressive extramedullary recurrence of IgD-λ-R/R MM. Patient 2 had been diagnosed with IgA-κ MM for 9 years; classic proteasome inhibitors and immunomodulators combined with traditional chemotherapy regimens had been used for many courses, but efficacy was poor and adverse drug reactions were unbearable. Patient 1 received a single injection of 4 × 10^6^/kg BCMA-7 × 19 CAR-T cells, and Patient 2 received a single injection of 3 × 10^6^/kg BCMA-7 × 19 CAR-T cells. The treatment scheme for the two patients was detailed in Figure 4B. The manufacturing of clinical grade CAR-T cells was successful in the two patients; the characterization of CAR-T cells, including phenotype and specific cytotoxicity, were shown in Table 2, Supplementary Figures 1, 2. The subjects received no other chemotherapy since enrolling in the study.

**Table 1:** Baseline characteristics.

**Characteristic**	**Patient**	
	**1**	**2**
Age (years)	69	62
Sex	Male	female
ECOG	1	0
Prior therapies	(1) VAD	(1) VAD
	(2) PAD	(2) PAD
	(3) PID	(3) VMD
		(4) Len+ DXM
		(5) Len+ DXM+ CTX
Prior lines of therapy	3	5
Relapsed/refractory status	Refractory second or higher line of therapy	Refractory third line of therapy
Primary diagnosis/sub-type	MM IgD-λ	MM IgA-κ
Durie-Salmon	IIIA	IA

*VAD, vincristine, doxorubicin, dexamethasone; PAD, bortezomib, doxorubicin, dexamethasone; PID, bortezomib, idarubicin, dexamethasone; CTX, Cyclophosphamide; Len, lenalidomide*.

**Table 2:** Characteristics of CAR-T products.

	**Specification**	**Patient 1**	**Patient 2**
Mycoplasma	Negative	Negative	Negative
Sterility (bacterial)^a^	Negative	Negative	Negative
Endotoxin (EU/mL)^a^	<3.5	<0.1	<0.1
CD3^+^%	>90%	99%	97.00%
CAR^+^ in CD3^+^%	>20%	71.20%	46.60%
CD4^+^ in CAR-T%	N/A	58.90%	26.80%
CD8^+^ in CAR-T%	N/A	39.00%	70.00%
T_SCM_ in CAR-T%	N/A	<0.1%	58.09%
T_CM_ in CAR-T%	N/A	97.40%	18.60%
CAR T cells/kg × 10^6^	N/A	4	3
CD8/CD4 CAR-T cell ratio	N/A	0.68	2.7
T cell expansion fold	N/A	350	500
Cancer cell killing activity	E: T = 5:1	92 ± 2.9%	92 ± 2.3%
Cell viability before transfection	>90%	97 ± 0.3%	98 ± 0.5%
Cell viability after transfection	>90%	98 ± 0.5%	96 ± 0.67%
Manufacturing time (days)	N/A	11	10

a *Results were not available at the time of infusion. E: T effect cell (CAR-T/Mock T): target cell (MM1S)*.

### Safety and Adverse Events (AEs)

AEs attributable to any cause occurring within 46 days of BCMA-7 × 19 CAR-T cell infusion were graded and evaluated according to CTCAE v5.0 (Table 3). Both patients received scheduled doses, and there was no dose-limiting toxicity. No serious adverse effects (>grade 3 AEs) were noted in either patient. AEs included maximum grade 2 and grade 1 events in patient 1 and patient 2, respectively, and neither patient experienced neurological symptoms (Table 3, Supplementary Table 1). Grade 1 CRS occurred in the two patients, according to Lee's (30) CRS grading criteria. The most common AEs related to BCMA-7 × 19 CAR-T cell infusion were neutropenia, thrombocytopenia, anemia, and fever. In addition to adverse hematological symptoms, both patients experienced grade 1 gastrointestinal bleeding and grade 1–2 hypoproteinemia. All AEs ranged from grade 1 to grade 3 in severity and resolved without special treatment. It is important to point out that AEs were self-limiting and reversible. Immunotherapy-induced changes in vital signs and kidney indicators, such as blood pressure, pulse, heart rate, respiration, CRP, uric acid and creatinine, were within the normal range and controllable (Supplementary Figure 3). Based on the safety profile of the two assessable DLT patients in phase 0, this regimen was deemed safe for study in phase 1.

**Table 3:** Adverse event grading based on CTCAE v5.0.

**Events**	**Patient 1**	**Patient 2**
**Any AE within 30 days of CAR-T infusion**		
Febrile neutropenia	0	1
Neutropenia	3	3
Anemia	2	2
Chest tightness	0	1
Thrombocytopenia	2	3
AST increased	1	0
Gastrointestinal bleeding	1	1
Low albumin	1	2
Fatigue	0	2
Hypocalcemia	1	1
Hyponatremia	1	1
Hypophosphatemia	1	1
Hypotension	1	0
Fever	2	0
Hypertension	0	2
Hyperglycemia	0	1
Appetite	0	1

*AST, Aspartate-aminotransferase; GGT, glutamyl-transpeptidase*.

### Clinical Response and Persistence

The two patients had an objective response within 1 month after BCMA-7 × 19 CAR-T cell infusion, with patient 1 achieving a VGPR of extramedullary recurrence and patient 2 attaining a CR (Figure 4A). As of April 12, 2020, the length of follow-up was 14 and 12 months of patient 1 and patient 2, respectively. Patient 1 was treated with lenalidomide plus dexamethasone for 11 months after diagnosis of extramedullary recurrence. A soft tissue mass on the fourth left rib progressed from 34 × 45 mm to 51 × 81 mm. Even after five courses of multi-drug chemotherapy, the soft tissue expanded to 72 × 110 mm (Figure 4D). A representative computed tomography (CT) scan showed a significant reduction in the size of the plasma cell tumor mass that maintained for more than 14 months (Figure 4D). Serum IgA, IgD, and IgM concentrations were within the normal range (Figure 4C). Patient 2 had undergone multiple courses of chemotherapy with poor efficacy, intolerable adverse reactions, and aggravation of thigh pain dependent on painkillers. After 2 months of CAR-T treatment, blood and urine concentrations of IgA and light chain protein decreased to regular base levels (Figures 4E,F).

We monitored plasma levels of seven cytokines before BCMA-7 × 19 CAR-T cell infusions and at multiple time-points after infusions. The peak fold increases over the baseline level were calculated for each cytokine for each patient (Figure 4G). The cytokines with the largest median fold increases were interferon-γ, IL-6 and IL-10. The cytokines with high peak blood levels are associated with CRS (35), and peripheral CAR^+^ cell levels are associated with anti-tumor responses in patients treated with CAR-T cells (37, 38). Ultimately, these data have demonstrated that BCMA-7 × 19 CAR-T cell therapy has potential clinical efficacy and durability with good safety.

## Discussion

To the best of our knowledge, this is the first preclinical and clinical study of anti-BCMA CAR-T cell secreting of IL-7 and CCL19 to treat R/R MM patients. The results are promising. First, 7 × 19 CAR-T cells targeting BCMA exhibited superior expansion, survival, accumulation of Tscm cells, migration and cytotoxicity compared to their traditional second-generation CAR-T counterpart. Second, the conditioning regimen of cyclophosphamide and fludarabine (day −4) followed by BCMA-7 × 19 CAR-T cells (day 0) at a dose of 4 × 10^6^ cells/kg was safe for further study and the toxicity was manageable. Finally, patients with R/R MM who received an infusion of BCMA-7 × 19 CAR-T cells responded effectively within 1 month and experienced no relapse in more than 12 months. The encouraging results facilitated the pivotal ongoing BCMA-7 × 19 phase 1 trial.

CAR-T cell immunotherapy is a significant milestone in modern cancer treatment. In the past 5 years, 125 clinical trials of CAR-T for R/R MM have been listed at https://clinicaltrials.gov, including 72 ones targeting BCMA. BB2121, a CAR-T cell therapy targeting BCMA, has received FDA breakthrough therapy approval and European Medicines Agency (EMA) priority approval. Neither VGPR nor CR was achieved in a low-dose group (50 × 10^6^ cells, *n* = 3); When the number of CAR-T cells increased to ≥150 × 10^6^ cells, the efficacy reached up to 94%, but this was accompanied by toxic hematological effects mostly of grade 3 or higher (39–41). It was recently reported that 6 of 15 patients who achieved complete remission experienced a relapse in just 6 months of follow-up (42). Overall, further improvements of CAR-T cell therapy in MM will be needed: optimizing the persistence and survival of CAR-T cells, decreasing the toxicity associated with CAR-T cell therapy, specifically targeting tumor cells and minimizing off-target toxicities.

Improving CAR persistence will rely heavily on understanding the biology of CAR-T cells and on the functionality and subsequent optimization of designs (43). Preclinical and clinical reports have indicated that 4-1BB co-stimulatory domain-containing CARs tend to persist better than those containing a CD28 co-stimulatory domain (44–48). Our BCMA-7 × 19 CAR vector contained a binding domain that recognizes BCMA, a CD8 transmembrane region, an intracellular 4-1BB co-stimulatory molecule, and a CD3ζ T cell signaling domain. IL-7 regulates the proliferation of T cells and maintains the stability of the intracellular environment (25), and CCL19 is a chemotactic agent for recruiting CCR7^+^ T cells and dendritic cells (22, 28, 49, 50). We showed the superiority of our BCMA-7 × 19 CAR-T cells in terms of proliferation and chemotaxis by calculating absolute cell counts and performing transwell migration assay, which provided a good start-up for solving the problems such as CAR-T cell proliferation, infiltration and accumulation in the tumor microenvironment.

Tscm are distinguishable from Tcm and Tem in phenotype, functional capacity to expand extensively, self-renewal, and differentiation potential (51–53). Several clinical studies have shown that the modifications to induce differentiation toward a Tcm/Tscm profile improve CAR-T cell response in subjects (54, 55). We confirmed a significant increase in the frequency of Tscm in BCMA-7 × 19 CAR-T cells by multiple cell surface marker analyses, which may be related to IL-7 in retaining the subpopulation of Tscm, compared to BCMA-hBBz CAR-T cells (56). In contrast, there was no difference in the frequencies of CD4^+^ and CD8^+^ CAR cells. Moreover, we successfully manufactured BCMA-7 × 19 CAR-T cells (Tscm + Tcm > 75%) for the first enrolled R/R MM patients, with one patient's Tscm up to 58%.

We have initiated a clinical trial to evaluate the safety and efficacy of BCMA-7 × 19 CAR-T cells in R/R MM patients. The first two enrolled and heavily treated R/R MM patients received autologous CAR-T cells (3–4 × 10^6^/kg) following lymphodepletion chemotherapy with cyclophosphamide (300 mg/m^2^ for 4 days) and fludarabine (30 mg/m^2^ for 4 days). Clinically significant toxicity was not observed, and most AEs were grade 1 or 2. Only two AEs (neutropenia and nausea) were grade 3 and were most likely related to cyclophosphamide/fludarabine. No high-grade AEs were recorded. None of the subjects experienced a DLT, and thus a maximum tolerated dose of CAR-T cells has not been determined yet.

In conclusion, this work preliminarily suggests that BCMA-7 × 19 CAR-T cells have substantial anti-MM activity and safety, although the small number of patients enrolled is a weakness of this study. Our study may help pave the way toward clinical application of BCMA-targeted fourth-generation CAR-T cells and thus highlight a potential strategy for dealing with malignancies of BCMA overexpression such as Waldenstrom macroglobulinemia and glioblastoma/astrocytoma (57, 58).

## Data Availability Statement

The raw data supporting the conclusions of this article will be made available by the authors, without undue reservation.

## Ethics Statement

The studies involving human participants were reviewed and approved by the institutional independent ethics committee of Lishui people's Hospital and Shunde Hospital, Southern Medical University. The patients/participants provided their written informed consent to participate in this study. The animal study was reviewed and approved by Wenzhou Medical University. Written informed consent was obtained from the individual(s) for the publication of any potentially identifiable images or data included in this article.

## Author Contributions

JG, ST, AZ, and DD: designed the study. DD, KW, AZ, and JG: performed the experiments, interpreted data, and wrote the manuscript. DF, CWe, JL, LL, SZ, XX, CWa, QH, and DL: performed experiments. YL, AZ, JJ, and BF: performed clinical trials. All authors contributed to the article and approved the submitted version.

## Conflict of Interest

AZ and JG were employed by Zhejiang Qixin Biotech. The remaining authors declare that the research was conducted in the absence of any commercial or financial relationships that could be construed as a potential conflict of interest.
