# Supplementary material for: The BCMA-Targeted Fourth-Generation CAR-T Cells Secreting IL-7 and CCL19 for Therapy of Refractory/Recurrent Multiple Myeloma
Source: Front Immunol. 2021 Mar 5;12:609421. doi: 10.3389/fimmu.2021.609421 (PMC7985831; doi:10.3389/fimmu.2021.609421)
Supplement: Supplementary file 1 [file Data_Sheet_1.PDF]

Supplementary Figures and Tables

Supplement Table 1. Adverse Event Grading Based on CTCAE v5.0

Adverse Events(AE[a])	Patient1	Patient2
CRS[b], Specific Symptomsb		
Rash	0	0
Fever	3	3
Nausea	0	2
Headache	0	0
Tachypnea	0	0
Tachycardia	0	0
Hypotension	1	0
Metabolic acidosis	0	0
Neurotoxicity, Specific Symptoms		
Encephalopathy	0	0
Tremor	0	0
Somnolence	0	0
Agitation	0	0
Aphasia	0	0
Delirium	0	0
Dizziness	1	0
Dyskinesia	0	0
Hallucination	0	0
Restlessness	0	0

[a]AEs labeled as possibly/probably/definitely related were considered as treatment-related AEs and not related or unlikely related as non-treatment-related AEs for this analysis. [b]CRS was graded per a modified grading system proposed by Lee et al[26].
Individual symptoms of CRS are graded per CTCAE, version 5.0.

Supplement Figure1

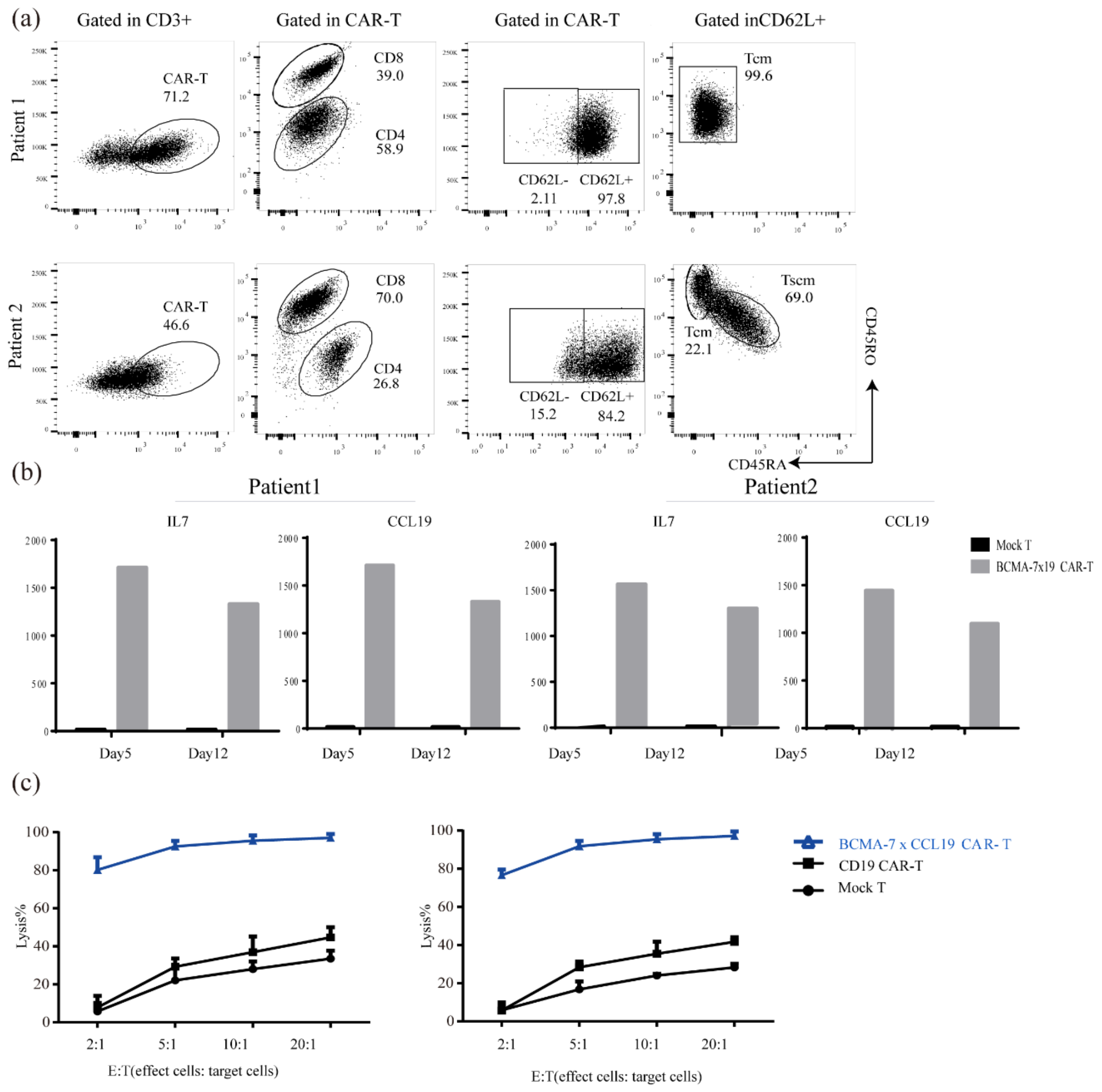

Supplemental Figure 1. BCMA-7x19 CAR-T cell manufacture for two subjects (A) CAR-T cell surface biomarkers detected by flow cytometry for CD3, CAR, CD62L, CD45RA, CD45RO expression. (B) Quantitative detection of secreted IL-7 and CCL19 by ELISA. Data represent the mean ± SD of triplicate wells. (C) CAR-T cells and target tumor cells were co-incubated for four hours at the indicated E:T ratios. Cytotoxicity assay was performed with MM1S-Luc-GFP cells as targets.

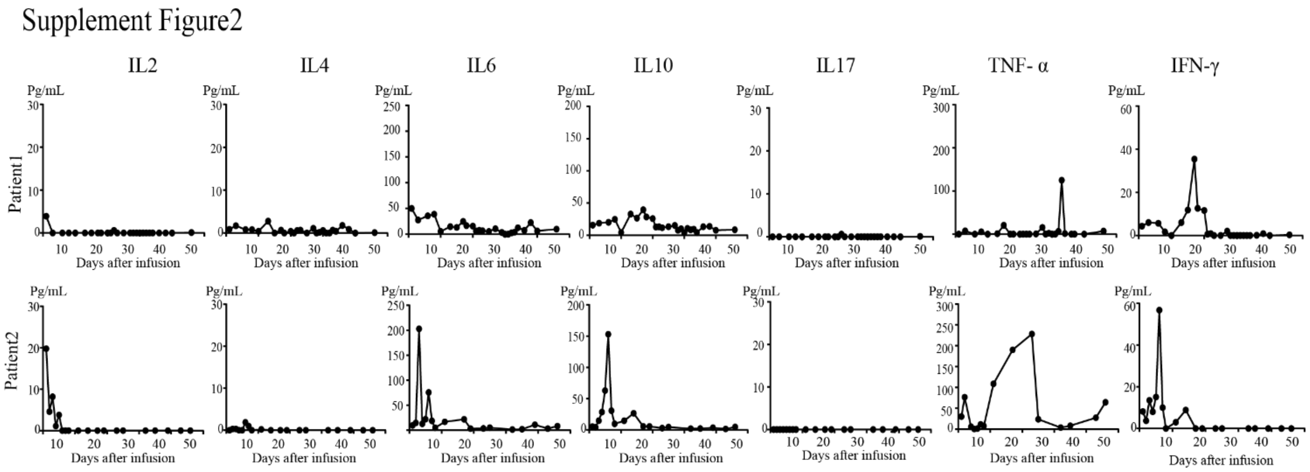

Supplemental Figure 2. Serum kinetics of a panel of cytokines in the two patients who received infusions with BCMA-7x19 CAR T cells (at 3-4×10^6/Kg CAR-T cells per patient), as determined by Luminex multiplex assay (R&D Systems).

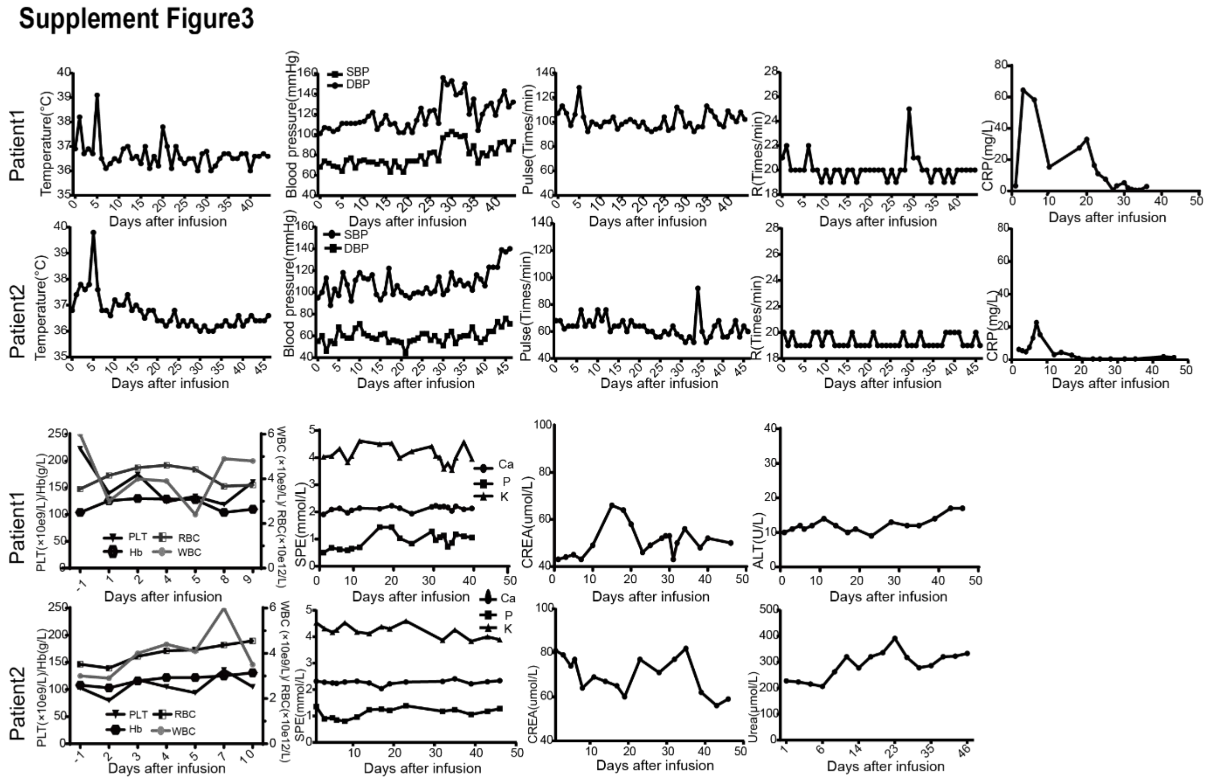

Supplemental Figure 3. vital signs (temperature, blood pressure, pulse heart rate, CRP, PLT, WBC, SPE, CREA, and ALT) in the two patients who received BCMA-7x19 CAR T cell infusions.
